# Serum Adiponectin Levels and Their Association With Cardiometabolic Risk Factors in Patients With Psoriasis

**DOI:** 10.7759/cureus.8128

**Published:** 2020-05-15

**Authors:** Wai Sze Agnes Chan, Choon Fong Liew, Colin Thiam Seng Theng, Hazel H Oon

**Affiliations:** 1 Dermatology, National Skin Centre, Singapore, SGP; 2 Diabetes & Endocrine Centre, Raffles Medical Group, Singapore, SGP; 3 Dermatology, Mount Alvernia Medical Centre, Singapore, SGP

**Keywords:** psoriasis, metabolic syndrome, cardiometabolic, adiponectin

## Abstract

Background and objective

Low adiponectin levels have been described in conditions with high cardiometabolic risk, including obesity, type 2 diabetes, insulin resistance, and hyperlipidaemia. Psoriasis is a chronic inflammatory skin disorder, and it is also associated with these conditions. In this study, we sought to assess the correlation between adiponectin levels and these risk factors including psoriasis severity. We investigated adiponectin value and its correlation with components of metabolic syndrome (MetS) and psoriasis severity.

Methods

Serum adiponectin levels were obtained from 215 psoriasis patients in a tertiary dermatology centre in Singapore. Psoriasis severity was measured with the psoriasis area and severity index (PASI), and cardiometabolic risk factors including obesity, hyperlipidaemia, insulin resistance, and waist circumference were measured. Patients answered a questionnaire regarding alcohol use, exercise, family history, smoking, and treatment history.

Results

Low adiponectin value was found in psoriasis patients with high body mass index (BMI) risk, high low-density lipoprotein (LDL), and low high-density lipoprotein (HDL). Patients with low HDL value had 25% lower adiponectin value compared to those with normal HDL. Adiponectin levels had a negative correlation with waist circumference. Psoriasis patients with MetS and a family history of cerebral vascular accidents (CVAs) had 17% and 18% lower adiponectin values than those without, respectively. There was no correlation between adiponectin level and PASI score.

Conclusion

Adiponectin levels were significantly decreased in psoriasis patients with obese-level BMI, MetS, increased abdominal girth, low HDL, high LDL, and a family history of CVA. Low adiponectin levels could play a role in predicting the development of MetS and possibly enable intervention to decrease the risk of cardiovascular mortality in psoriatic patients.

## Introduction

Psoriasis is a chronic inflammatory condition, which is mediated by the interplay between the innate and adaptive immune systems [1]. Its pathogenesis is still not fully understood. It not only causes disease of the skin and joints, but also systemic inflammation, and is associated with an increased prevalence of metabolic disorders such as central obesity, dyslipidaemia, hypertension, and insulin intolerance [2]. Both psoriasis and metabolic syndrome (MetS) are characterised by an inflammatory T cell-mediated process, with over-expression of pro-inflammatory cytokines, tumour necrosis factor (TNF)-a, and interleukin (IL)-6 [3].

Adiponectin is an adipocyte-specific secretory protein, and it is abundant in normal circulation. It is a collagen-like protein that regulates the metabolism of lipids and glucose [4]. Adiponectin acts to activate multiple signaling pathways, which mediate anti-inflammatory, insulin-sensitizing, and anti-atherogenic functions via the suppression of TNF-a and IL-6. Decreased adiponectin level has been associated with obesity, type 2 diabetes, insulin resistance, and hypoadiponectinaemia, all of which are associated with MetS [5]. A recent review suggested that adiponectin could have a central role in the pathogenesis of MetS, with adiponectin levels associated with MetS risk independent of insulin resistance and obesity [6].

In Singapore, the prevalence of MetS has been reported to range between 17.7-26.2%, depending on criteria adopted [7]. MetS is a strong predictor of cardiovascular disease, diabetes, and stroke, with a reported three-fold increase in cardiovascular mortality associated with MetS. Adiponectin has also been reported to have a negative correlation with body mass index (BMI) and psoriasis area severity index (PASI) scores in psoriatic patients [8-10]. We sought to examine the correlation between adiponectin levels in a Singaporean population with psoriasis. We also sought to assess the correlation of adiponectin levels with psoriasis severity and MetS.

## Materials and methods

The study was approved by the local institutional review board (IRB). All participants were enrolled after having signed the IRB-approved written informed consent.

Patients

We enrolled participants with psoriasis (n=338) who attended the outpatient dermatology clinic at the National Skin Centre, a tertiary referral centre in Singapore, between October 2007 and February 2009. Inclusion criteria were as follows: patients aged between 18-69 years with a clinical diagnosis of chronic plaque psoriasis. Patients who were unable to give consent and pregnant women were excluded from this study. Serum adiponectin levels were obtained (n=215). Fasting serum samples were obtained for glucose and lipid profiles. Weight and height (in metres) were measured using calibrated digital weighing scales and stadiometer, respectively. The waist and hip circumference were measured with a measurement tape; the waist was defined as the narrowest circumference between the iliac crest and costal margin, and hip as the widest circumference between the waist and thigh. Trained staff conducted all measurements. The BMI was calculated as the bodyweight of participants divided by the square of the height. A BMI of <23 was considered healthy, that of 23.1-27.4 was seen as overweight, and >27.5 was considered obese. Blood pressure was measured using a standard mercury sphygmomanometer, taken on two separate visits. Psoriasis disease activity was measured using the PASI at the time of enrolment by the attending dermatologist, and information on medication was extracted from the electronic medical records. MetS was diagnosed based on three or more of the following National Cholesterol Education Program-Adult Treatment Panel III (NCEP-ATP III) criteria: waist circumference of ≥90 cm in men or ≥80 cm in women; hypertriglyceridaemia of ≥1.7 mmol/L; high-density lipoprotein (HDL) cholesterol of ≤1.03 mmol/L in men or ≤1.29 mmol/L in women; blood pressure of ≥130/85 mmHg; fasting plasma glucose of ≥5.6 mmol/L [11].

A questionnaire containing questions about ethnicity, age, psoriasis duration and self-reported diagnosis of diabetes, hypertension, hyperlipidaemia, ischaemic heart disease, family history of ischaemic heart disease, family history of psoriasis, family history of cerebral vascular accident (CVA), and lifestyle habits of smoking, alcohol and physical exercise was used.

Laboratory investigation

Serum cholesterol and triglycerides were measured with enzymatic procedures. Adiponectin levels (μg/ml) were analysed by ELISA-based assays. Plasma glucose was measured using a glucose oxidase method.

Statistical analysis

Data were entered and analysed using Stata version 13 (StataCorp, College Station, TX). Bivariate analysis using Kruskal-Wallis tests was done to compare the difference in adiponectin levels between different groups or levels of components of MetS and PASI score separately. Non-parametric methods were used, as the adiponectin values were not normal-distributed. A p-value of <0.05 was considered statistically significant.

To analyse the relationship of adiponectin levels with the severity of psoriasis, components of MetS, and other potential baseline characteristics, a linear regression model was built on the log-transformed adiponectin values as the adiponectin values had a right-skewed distribution. Exp (Coef.) of the model showed the percentage of change in adiponectin values for a unit change in each baseline characteristic, holding others constant.

## Results

A total of 338 patients were enrolled in the study. Serum level of adiponectin was obtained from 215 patients; 154 were male (71.6%), and 61 were female (28.4%). Of these, 153 (71.6%) were Chinese, 23 (10.7%) were Malay, and 39 (18.1%) were Indian. The median age was 50.1 years (range: 19.4-69.3). Table 1 shows the baseline characteristics of psoriasis patients with adiponectin values.

**Table 1:** Baseline characteristics of psoriasis patients with adiponectin values BMI: body mass index; PASI: psoriasis area severity index; HDL: high-density lipoprotein; LDL: low-density lipoprotein; SD: standard deviation

Characteristics	Psoriasis patients with adiponectin values (n=215)
Age in years, mean ± SD	49.2 ± 11.0
Male, n (%)	154 (71.6%)
Female, n (%)	61 (28.4%)
Chinese, n (%)	153 (71.6%)
Malay, n (%)	23 (10.7%)
Indian, n (%)	39 (18.1%)
Mean adiponectin, μg/ml, mean ± SD	2.7 ± 1.3
Elevated blood pressure, n (%)	135 (62.8%)
BMI, mean ± SD	26.1 ± 5.1
Low BMI obesity risk (<23), n (%)	60 (27.9%)
Moderate BMI obesity risk (23-27.4), n (%)	81 (37.7%)
High BMI obesity risk (≥27.5), n (%)	74 (34.4%)
Waist circumference, cm, mean ± SD	90.1 ± 15.2
Abdominal obesity, n (%)	128 (59.5%)
Triglyceride level, mmol/L, mean ± SD	1.5 ± 0.8
Hypertriglyceridaemia, n (%)	63 (29.4%)
High HDL, mmol/L, mean ± SD	1.2 ± 0.3
Low HDL, n (%)	75 (35.0%)
LDL level, mmol/L, mean ± SD	3.3 ± 0.8
Impaired fasting glucose, n (%)	59 (27.4%)
Metabolic syndrome, n (%)	97 (45.1%)
Duration of psoriasis in years, mean ± SD	15.1 ± 9.8
PASI, mean ± SD	9.4 ± 8.1
PASI <10, n (%)	149 (69.6%)
PASI ≥10, n (%)	65 (30.4%)
Smoker, n (%)	93 (43.3%)
Alcohol use, n (%)	96 (44.7%)
Exercise, n (%)	117 (54.4%)
Arthritis, n (%)	82 (38.1%)
Diabetic, n (%)	32 (14.9%)
History of ischaemic heart disease, n (%)	18 (8.4%)
History of cerebrovascular accident, n (%)	6 (2.8%)
Family history of psoriasis, n (%)	58 (27.1%)
Family history of ischaemic heart disease, n (%)	52 (24.3%)
Family history of cerebrovascular disease, n (%)	53 (24.8%)

BMI obesity was associated with a decrease in adiponectin levels (Table 2). Mean adiponectin value was 3.3 μg/ml in low-risk healthy BMI (<23), 2.5 μg/ml in moderate BMI (23.1-27.4), and 2.4 μg/ml in high-risk BMI (≥27.5). After adjusting for other baseline characteristics, including PASI score, smoking, age, and MetS, obese patients had 18% lower adiponectin values than patients with a healthy weight (p=0.02).

**Table 2:** Test difference in adiponectin values between different groups or components of metabolic syndrome PASI: psoriasis area severity index; BMI: body mass index; HDL: high-density lipoprotein; SD: standard deviation

Components of metabolic syndrome	Mean adiponectin value, μg/ml, mean ± SD	Test of difference (p-value)
PASI <10	2.6 ± 1.2	0.37
PASI ≥10	2.9 ± 1.4	
Elevated Blood pressure	2.8 ± 1.3	0.66
Normal blood pressure	2.7 ± 1.2	
BMI <23 (healthy weight)	3.3 ± 1.6	<0.01
BMI 23-27.4 (overweight)	2.5 ± 1.0	
BMI ≥27.5 (obese)	2.4 ± 1.1	
Abdominal obesity absent	3.2 ± 1.4	<0.01
Abdominal obesity present	2.4 ± 1.0	
Hypertriglyceridemia absent	2.8 ± 1.3	0.15
Hypertriglyceridemia present	2.5 ± 1.1	
Low HDL	3.0 ± 1.3	<0.01
Normal HDL	2.2 ± 0.9	
Impaired fasting glucose	2.7 ± 1.3	0.20
Normal fasting glucose	2.6 ± 1.3	
Metabolic syndrome present	2.9 ± 1.4	<0.01
Metabolic syndrome absent	2.4 ± 1.0	

Mean adiponectin level for low HDL was 2.2 μg/ml compared to 3.0 μg/ml for normal HDL. Patients with low HDL had 25% lower adiponectin value compared to those with normal HDL (p<0.01). Adiponectin levels were decreased in patients with high BMI risk, high LDL, and low HDL. Bivariate analysis demonstrated that adiponectin was significantly decreased in patients with MetS. Mean adiponectin level was 2.9 μg/ml in those without MetS, compared to 2.4 in those with MetS (Table 2).

**Figure 1:**
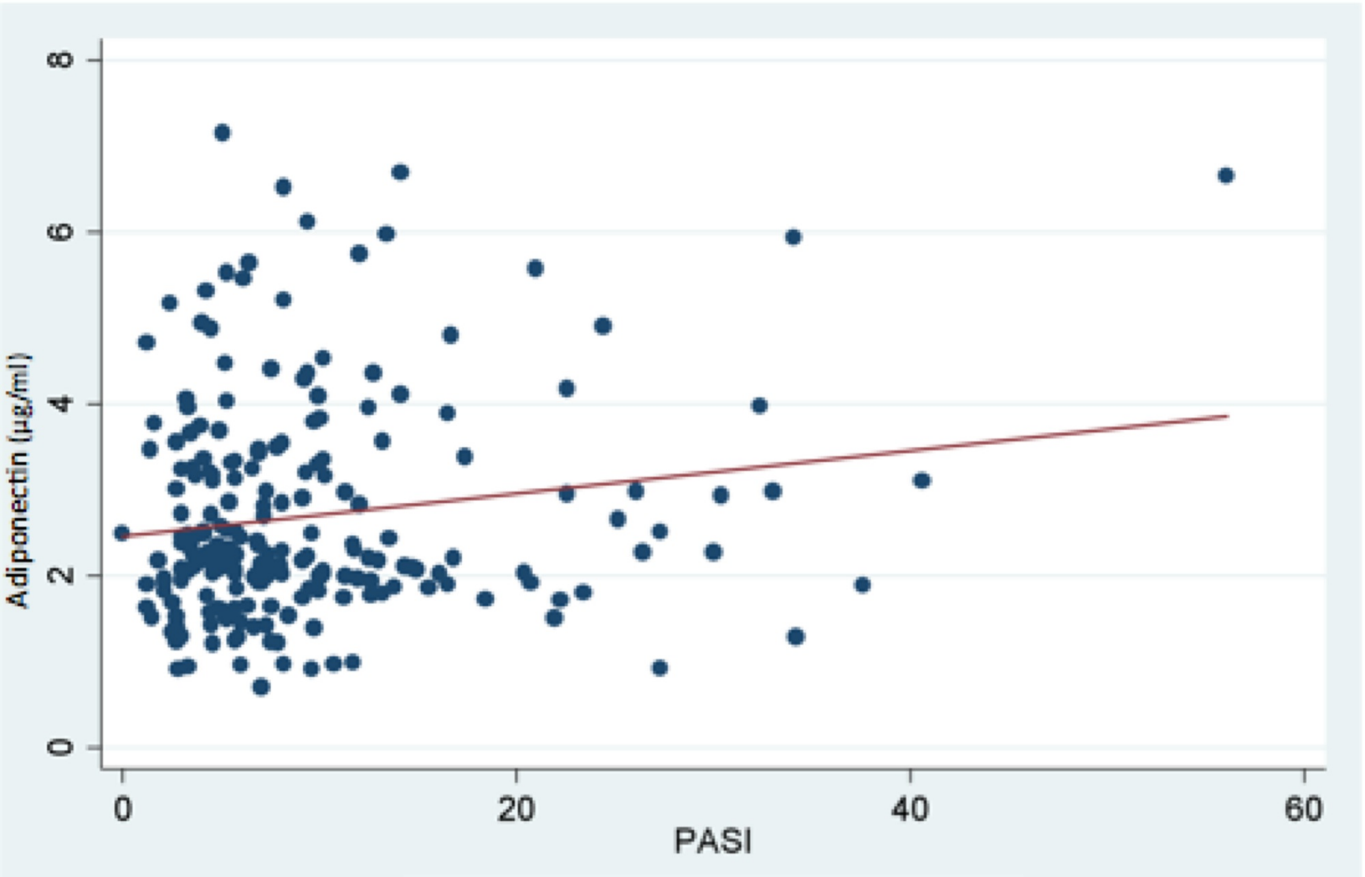
Chart showing no correlation between adiponectin value and PASI score PASI: psoriasis area severity index

After adjusting for other potential baseline characteristics such as PASI score, smoking, and age, patients with MetS had 17% lower adiponectin value than those without (Table 2). Age and family history of CVA were also significantly correlated with lower adiponectin levels after adjusting for other baseline characteristics. Patients with a family history of CVA had 18% lower adiponectin value than patients without family history of CVA. However, there was no correlation between adiponectin value and PASI score (p=0.29; Figure 1).

**Figure 2:**
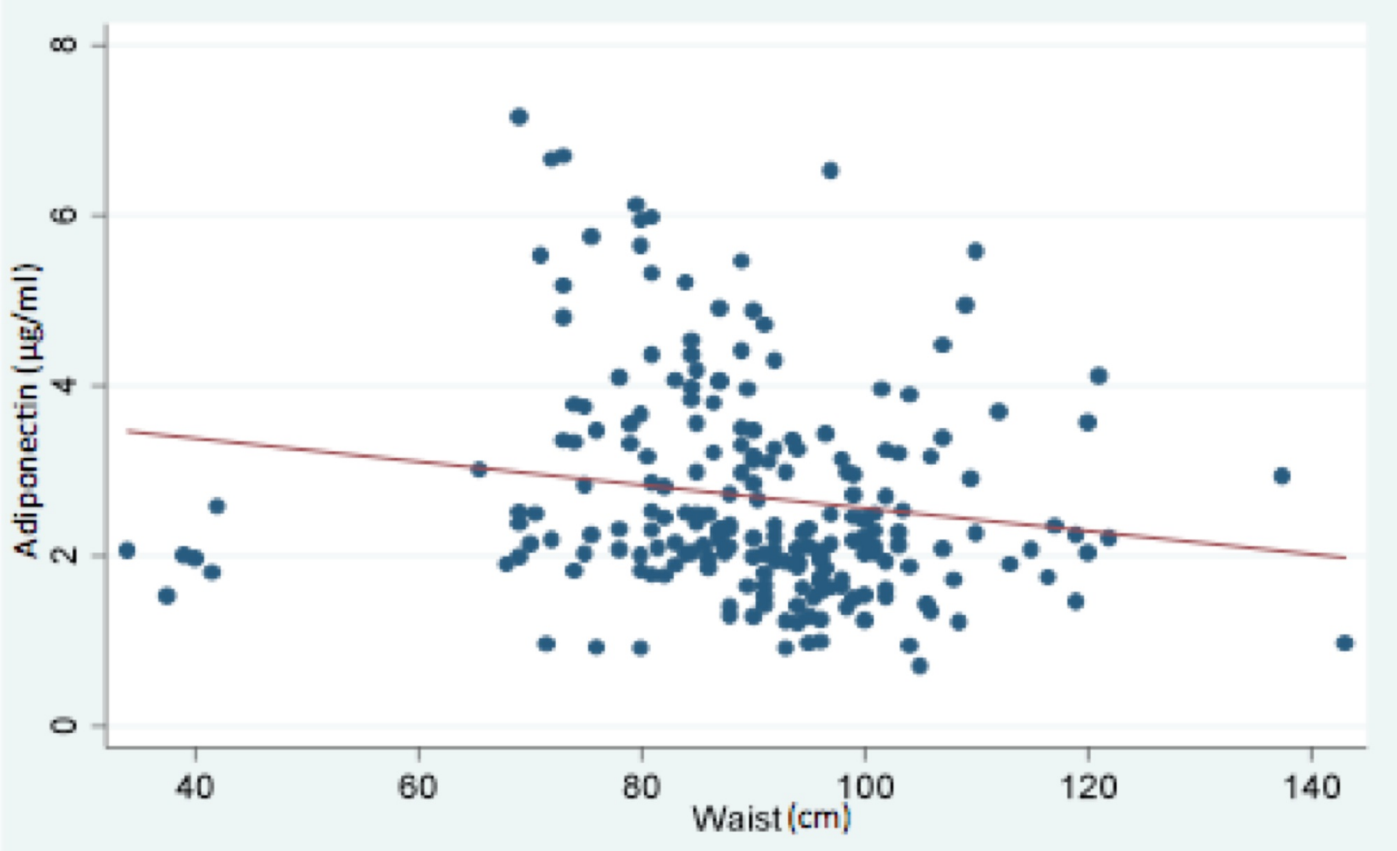
Chart showing negative correlation between adiponectin value and abdominal obesity

Abdominal obesity was associated with a significant low adiponectin level and demonstrated by a negative correlation between adiponectin level and waist circumference. The mean adiponectin level was 3.3 μg/ml in those with healthy waist circumference compared to 2.4 μg/ml in patients with abdominal obesity (Figure 2).

## Discussion

Psoriasis is a chronic inflammatory, immune-mediated skin disease, characterised by T helper cell dysfunction with over-expression of pro-inflammatory cytokines. The same pro-inflammatory cytokines, including TNF-a and IL-6, have been implicated in adipose tissue dysfunction and insulin resistance in central obesity [12]. Although the exact pathogenesis of psoriasis is still not fully defined, high BMI is known to be an important risk factor [13]. This is in keeping with the prevalence of overweight and obese patients in our study, with only 27.8% normal-weight patients, while 54.2% were overweight or pre-obese and 17.9% obese.

Adiponectin is a cytokine secreted by adipocytes and is present at relatively high concentrations. It has anti-inflammatory effects and has been shown to play an important role in lipid metabolism and atherogenesis, and provides protection against metabolic and cardiovascular diseases [14]. Adiponectin suppresses the expression of TNF-a, IL-6, and interferon-g; improves insulin sensitivity; and repairs damaged vasculature [15]. It has been established that circulating adiponectin concentrations decrease with increasing levels of obesity [16]. Studies have also reported a decrease in adiponectin value in psoriasis patients compared to normal controls [10]. Our study showed consistent findings with an 18% lower adiponectin level in those with obese BMI (>27.5) compared to those with healthy BMI. Low HDL was also associated with a 25% lower adiponectin level compared to normal HDL levels in psoriatic patients.

The link between adiponectin and inflammation has been reported to be a vicious loop, with obesity being associated with inflammation in adipose tissue. Pro-inflammatory factors suppress adiponectin suppression, low levels of adiponectin increase insulin resistance and risk of cardiovascular disease, and low levels of adiponectin promote inflammation, thus generating a self-sustaining inflammatory loop [17]. Prospective studies have shown an increased risk of cardiovascular events in patients with low adiponectin levels [18]. Furthermore, in psoriasis, the chances of MetS and cardiovascular diseases are also increased and independently associated with the disease, further increasing the risk of cardiometabolic complications.

MetS is a state of chronic systemic inflammation requiring at least three of the following five pathophysiological states: abdominal obesity, impaired glucose regulation, hypertriglyceridaemia, reduced HDL, and hypertension. Those with MetS have a strong propensity for cardiovascular mortality (with a three-fold increase reported in Singapore), diabetes, and coronary heart disease. Many studies have shown that the prevalence of individual features of MetS is much more prominent in the psoriatic population. The encoding gene for adiponectin is located in the same susceptibility region as genes involved in MetS, cardiovascular disease, and type 2 diabetes mellitus (DM) [19,20]. Our study illustrates the high incidence of MetS (45.1%) in a Singaporean population with psoriasis. Referral bias is a possibility as our centre is a tertiary centre. BMI has been reported to be a risk factor for psoriasis, which is also a component for MetS. Our data is consistent with other published data, with a 17% decrease in adiponectin levels in those with MetS compared to those without. A significant decrease of 18% adiponectin level was also demonstrated in patients with a family history of CVA compared to those without. This highlights the importance of the family history of CVA and perhaps the role it plays in genetic predisposition to increased risk of cardiovascular and metabolic diseases.

A recent review demonstrated that adiponectin levels were associated with MetS risk independent of insulin resistance and obesity and could play a central role in the etiology of MetS [6]. Another study found that psoriasis is associated with decreased plasma adiponectin levels independently of cardiometabolic risk factors [21]. Psoriasis may have a unique effect on adiponectin signaling, leading to pro-inflammatory features of psoriasis independent of obesity. Diminished adiponectin leads to a more pro-atherogenic phenotype, due to the loss of inhibition of monocyte cell adhesion, vascular adhesion molecule 1, endothelial-leukocyte adhesion molecule-1, and transformation of macrophages to foam cells, all of which lead to the formation of atherosclerotic plaque formation [15]. The finding of decreased adiponectin levels in psoriasis patients with MetS, obese BMI, high LDL, and low HDL in our study signify underlying adipose tissue dysfunction and highlight the increased cardiometabolic risk. In addition to promoting healthy BMI and monitoring of lipid, monitoring of adiponectin levels may play a role in early identification of metabolic risk. In our study, the mean adiponectin value was 2.7 μg/ml. Low adiponectin levels could be a future biomarker for assessment of cardiovascular risk in psoriasis, and further large prospective studies with controls are needed to assess this.

Adiponectin levels have been shown to increase after successful anti-inflammatory treatment in psoriasis and reduction of BMI [22]. Studies using statins in the treatment of patients with hyperlipidaemia, obesity, diabetes, and healthy controls have shown to increase adiponectin levels. Pitavastatin has been shown to consistently increase plasma adiponectin, and it does not lead to new-onset or statin-induced diabetes either [23,24]. Perhaps, statin therapy should be considered earlier in psoriatic patients with low adiponectin values.

Our study showed no correlation between adiponectin and PASI score. Other studies have shown a negative correlation with PASI score [9,10]. It is postulated that inflammation leads to an increased release of TNF-a and inhibition of adiponectin. Patients in our study had a relatively mild psoriatic disease, with 69.6% having a PASI score of less than 10, and perhaps this played a part in the lack of correlation, as a low PASI score indicates low disease activity with low TNF-a levels.

## Conclusions

Adiponectin levels were significantly decreased in psoriasis patients with obese BMI, MetS, low HDL, high LDL, and a family history of CVA. There was no correlation between adiponectin level and PASI score. Recent studies have shown that adiponectin may play a central role in the pathogenesis of MetS independent of insulin resistance and obesity. Weight reduction, improved blood pressure control, and improved HDL have been shown to increase adiponectin levels. A higher level of adiponectin has been associated with a decrease in the risk of diabetes and coronary heart disease. Hence, low adiponectin levels could play a role in predicting the development of MetS and possibly enable intervention to decrease the risk of cardiovascular mortality in psoriatic patients. Physicians should encourage patients to address and decrease their modifiable cardiovascular risk factors. Further studies are needed to assess the functional use of adiponectin as a biomarker for assessment and early intervention to decrease cardiometabolic complications.
